# Terahertz spectroscopy of diabetic and non-diabetic human blood plasma pellets

**DOI:** 10.1117/1.JBO.26.4.043006

**Published:** 2021-02-12

**Authors:** Anastasiya A. Lykina, Maksim M. Nazarov, Maria R. Konnikova, Ilia A. Mustafin, Vladimir L. Vaks, Vladimir A. Anfertev, Elena G. Domracheva, Mariya B. Chernyaeva, Yuri V. Kistenev, Denis A. Vrazhnov, Vladimir V. Prischepa, Yulia A. Kononova, Dmitry V. Korolev, Olga P. Cherkasova, Alexander P. Shkurinov, Alina Y. Babenko, Olga A. Smolyanskaya

**Affiliations:** a Institute of Photonics and Optical Information Technologies, ITMO University, Saint-Petersburg, Russia; b National Research Center “Kurchatov Institute,” Moscow, Russia; c Institute on Laser and Information Technologies, Russian Academy of Sciences — Branch of Federal Scientific Research Center “Crystallography and Photonics,” Russian Academy of Sciences, Moscow, Russia; d Department of Physics, Lomonosov Moscow State University, Moscow, Russia; e Ioffe Institute, Saint-Petersburg, Russia; f Institute for Physics of Microstructures, Russian Academy of Sciences, Nizhny Novgorod, Russia; g Tomsk State University, Tomsk, Russia; h Institute of Strength Physics and Materials Science, Siberian Branch of the Russian Academy of Sciences, Tomsk, Russia; i Almazov National Medical Research Centre, Saint-Petersburg, Russia; j Institute of Laser Physics, Siberian Branch of the Russian Academy of Sciences, Novosibirsk, Russia

**Keywords:** terahertz time-domain spectroscopy, blood plasma, diabetes, lyophilization, pellets, principal component analysis

## Abstract

**Significance:** The creation of fundamentally new approaches to storing various biomaterial and estimation parameters, without irreversible loss of any biomaterial, is a pressing challenge in clinical practice. We present a technology for studying samples of diabetic and non-diabetic human blood plasma in the terahertz (THz) frequency range.

**Aim:** The main idea of our study is to propose a method for diagnosis and storing the samples of diabetic and non-diabetic human blood plasma and to study these samples in the THz frequency range.

**Approach:** Venous blood from patients with type 2 diabetes mellitus and conditionally healthy participants was collected. To limit the impact of water in the THz spectra, lyophilization of liquid samples and their pressing into a pellet were performed. These pellets were analyzed using THz time-domain spectroscopy. The differentiation between the THz spectral data was conducted using multivariate statistics to classify non-diabetic and diabetic groups’ spectra.

**Results:** We present the density-normalized absorption and refractive index for diabetic and non-diabetic pellets in the range 0.2 to 1.4 THz. Over the entire THz frequency range, the normalized index of refraction of diabetes pellets exceeds this indicator of non-diabetic pellet on average by 9% to 12%. The non-diabetic and diabetic groups of the THz spectra are spatially separated in the principal component space.

**Conclusion:** We illustrate the potential ability in clinical medicine to construct a predictive rule by supervised learning algorithms after collecting enough experimental data.

## 1 Introduction

Diabetes mellitus is a disease caused by the deficit or reduced efficiency of endogenous insulin resulting in blood sugar imbalance.^1^ The development of diabetes is associated with impairments in the carbohydrate, protein, and lipid metabolism. It is almost always accompanied by a significant increase of the blood concentrations of glucose, corticosteroid hormones, and some other metabolites.^1^ Protein glycation is a non-enzymatic reaction between the carbonyl groups of monosaccharides (e.g., glucose and fructose) and amino groups of proteins (e.g., albumin). Glycation initiates a cascade of protein modifications resulting in the loss of both secondary and tertiary protein structures.^2^ Currently, the following tests are used in clinical practice to diagnose diabetes mellitus and monitor patients, for example, determining the level of glucose in venous blood plasma by the enzyme method or in capillary blood by an electrochemical method, as well as determining the level of glycated hemoglobin in whole venous blood by liquid chromatography.^3^^,^^4^ All these methods require not only expensive equipment, but also reagents for their implementation. In addition, some studies can only be performed with fresh blood that cannot be stored for a long time. However, to determine some parameters, blood can be stored for a long time, but bulky freezers are required to store it. The development of methods for determining blood parameters and for storing biological samples will reduce the cost of performing diagnostic tests.^3^^,^^4^

Terahertz (THz) radiation and technologies based on it have been actively developed and increasingly used in many fields of science and technology,^5^^,^^6^ including medical diagnostics and therapy.^7^^–^^9^ In last five years, THz spectroscopy has been demonstrated as a tool for highly accurate measurement of even small quantities of sugar molecules in liquid solutions and the selective identification of different sugar molecules. In terms of biochemical analysis, THz spectroscopy can be used for quantitative determination of the global hydration state of sugar solutions.^10^ The measured hydration number correlates with the number of carbonyl groups in the sugar solution. In addition, this number inversely depends on the concentration of dissolved compound due to the overlapping of hydration shells and dipole correlation functions of the solution. Albumin incubation (the main blood plasma protein) with fructose is accompanied by the formation of covalent bonds between the sugar carbonyl groups and protein amino groups,^2^ which decreases the portion of fructose molecule with associated water molecules. In other words, the amount of free water molecules increases after albumin incubation with fructose.^11^

With regard to diabetes diagnosis, THz spectroscopy examines whole blood and blood plasma of human beings and animals, some liquid models of diabetes, such as aqueous solutions of glycated albumin or hemoglobin, as well as sugar solutions such as glucose, sucrose, galactose, and other sugars dissolved in different concentrations in water. The experimental data on the dependence of the absorption coefficient in the THz frequency range on the concentration of various sugar solutions in the literature are contradictory. On the one hand, one group of authors claims that the absorption coefficients and refractive index of sugar solutions decrease with increasing sugar concentration. This effect is caused by the partial replacement of free water, which strongly absorbs THz radiation, by a component that absorbs less in this frequency range, namely glucose.^12^^,^^13^ This tendency was demonstrated with such objects: dry sucrose and water solutions,^14^,^15^ albumin incubated with sugars (glucose or fructose),^11^^,^^16^^,^^17^ glucose aqueous solution,^10^,^17^^,^^18^ monosaccharide (glucose and fructose), and disaccharide (sucrose and trehalose),^10^ fructose and D-glucose,^19^ rat blood plasma with and without diabetes,^20^^,^^21^ human blood plasma with and without diabetes,^20^ and *in vivo* human palm skin.^22^

On the other hand, the other group of authors states that the absorption coefficients and refractive index of sugar solutions increase with increasing sugar concentration. In Refs. 19, 23, and 24, it was demonstrated that the absorption intensities increase of both aqueous glucose and fructose solutions with the concentrations increasing, indicating clearly the THz absorption spectra of these solutions are affected by the amount of glucose or fructose molecules involved. The same tendency was observed for human blood plasma with and without diabetes^24^ and on *in vivo* ear capillaries of a diabetic mouse.^25^

This difference between the experimental data of various authors can be associated with the type of sugar–protein mixture that is (1) glycated protein or (2) mixture of two substances (the effective medium model). Glycation the protein with sugar (first case) demands using high temperature, long-term incubation, and buffer. Glycation is accompanied by the formation of covalent bonds between the sugar carbonyl group and protein amino groups, which decrease the portion of sugar molecules with associated water molecules. Then the amount of free water molecules increases and absorption coefficients of these solutions increase.^13^ In the mixtures of glucose and protein, without its glycation (second case) results on partial replacement of free water by a glucose that absorbs less than water in THz frequency range.^18^ In this case, the absorption coefficient of sugar solution decreases. This difference between the experimental data of various authors can also be associated with the features of the experimental schemes of THz spectrometers, different data processings, or with equipment or samples calibration. Despite inconsistent data, it can be claimed that THz radiation can be used in the high-precise measurement of sugar levels. For example, it was found out that the THz absorption coefficients and the blood glucose levels perform a linear relationship.^24^ This linear correlation indicates that quantitative blood glucose level analysis is feasible using THz time-domain spectroscopy (THz-TDS).^24^

Previous research in THz spectroscopy has encountered difficulties in diagnosing and storing the liquid whole blood or blood plasma samples. This may also be the cause of the inconsistent data reported in the literature. Research on tableted samples can solve this problem. Our previous study has shown^26^ that from the point of view of spectroscopic studies, the tableted samples absorb practically no moisture and have low absorption in the THz frequency range. Moreover, they can be conveniently fixed in a vertical holder for THz-TDS in transmission mode. Since pellets are the pressed tablets from small-fractional crystals from triglycerides, albumin, and fibrinogen, their surface contains certain roughness and their internal composition is characterized by spatial inhomogeneities of the refractive index. At the same time, they are relatively uniformed and quasi-flat to effectively transmit THz radiation and can be used in THz holographic measurements to obtain a spatially resolved distribution of optical properties providing statistically reliable results. For storing samples in biobanks in medical institutions, pellet samples also have a number of advantages. Thus the size of a pellet can be hundred times smaller than an Eppendorf. They can be examined several times, stored at room temperature, and be more transportable.

The main idea of this work is to propose a method for diagnosis and storing the samples of diabetic and non-diabetic human blood plasma and to study these samples in the THz frequency range. There are several technologies for removing water from biological samples. One of them is lyophilization for liquid samples at high pressure.^27^^,^^28^ The lyophilization method allows getting dry tissue without losing the structural integrity and biological activity of the sample. During lyophilization, free water is removed from the blood plasma. The hydrogen bonds between the protein and water are replaced by the covalent bonds between the carbonyl groups of glucose and the amino groups of the protein.^27^^–^^29^ This method of preparing the studied objects allows their repeated study and transportation to various research laboratories. Samples of human blood plasma with type 2 diabetes mellitus and a conditionally healthy participants were used as the studied objects. The lyophilization of these liquid objects and their pressing into pellets were performed. To avoid the inconsistency found in the literature, the experimental studies in this work were conducted on two different THz-TDS, with an approximately similar experimental schemes, and we calculated the optical properties (absorption coefficient and refractive index) using the same equations. Moreover, the study of the samples in different laboratories can improve the accuracy and objectivity of the results. To classify non-diabetic and diabetic groups’ spectra, a diagnostic model based on the THz properties of the studied samples was developed using multivariate statistics.

## 2 Experiments and Methodology

### 2.1 Method for the Preparation of Blood Plasma Pellets

Venous blood from patients with type 2 diabetes mellitus and conditionally healthy participants was collected in the Endocrinology Department of Almazov National Medical Research Centre. This center provides medical care for diabetic patients. Three patients and two participants were male, age-matched (39- to 43-year old) people. All experimental protocols used in this investigation were reviewed and approved by the patients, participants, and the Use Commission of the Medical Centre. Venous blood was collected in the morning after 8 to 12 h of fasting in a tube with the anticoagulant $K_{3}\mathrm{EDTA}$ (Vacutest Kima, Italy). Plasma was obtained for the analysis of biochemical parameters by centrifugation of whole blood at 3000 rpm for 15 min in a laboratory centrifuge (Eppendorf 5702R, Germany) at a temperature of +4°C. Values of biochemical parameters of blood plasma samples and reference intervals are presented in Table 1 (the level of glycated hemoglobin was obtained on the whole blood). These parameters were determined by the enzyme method, immunonephelometric method, kinetic colorimetric method, colorimetric method, and high-yield liquid chromatography. Table 1 demonstrates that the concentration of glucose, triglycerides, and glycated hemoglobin in the samples of a patient with diabetes increases 1.5, 2.0, and 2.3 times, respectively.

**Table 1:** Biochemical parameters levels in blood plasma samples.

Biochemical parameter	Non-diabetic sample	Diabetic sample	Reference interval	Measurement method
Albumin (g/l)	49.10	45.20	34 to 48	Immunonephelometric method
Glucose (mmol/l)	4.34	6.51	3.30 to 6.10	Enzyme method
Triglycerides (mmol/l)	0.82	1.69	<1.77	Enzyme method
Glycated hemoglobin (%)	4.8	11.0	4 to 6	High-yield liquid chromatography
Bilirubin (mmol/l)	0.027	0.007	0.003 to 0.020	Colorimetric method
Creatinine (mmol/l)	0.08	0.06	0.06 to 0.10	Kinetic colorimetric method
Total cholesterol (mmol/l)	4.49	3.43	3.50 to 5.00	Enzyme method
Uric acid (mmol/l)	0.25	0.15	0.20 to 0.42	Colorimetric method

Human blood plasma samples were frozen at a temperature of −80°C (low-temperature refrigerator DW-86L388A, Haier, China). Then it was lyophilized by freeze-drying VaCo 2 (ZirBus, Germany) at a temperature of −50°C and a pressure of 3 Pa. Freezing was performed before lyophilization, since during lyophilization under the influence of high pressure, blood plasma components can be destroyed. Dried blood plasma was a sponge consisting of biological crystals. The sponge was destroyed by a metal spatula and crushed to crystals with a size of several tens of micrometers. The use of a mortar and pestle was impossible, since grinding the proteins in the composition of the samples would lead to their unwanted adhesion and the formation of round granules.

The lyophilized plasma powder was weighed (analytical balance OHAUS Discovery, Switzerland) and then placed in a steel press mold. Using the laboratory presses (Enkor, Russia and Specac, UK) at a certain moulding pressure, the blood plasma pellets were obtained. Each crystal of pellets contains a certain percentage of lipids (triglycerides), proteins (albumin), and fibrinogen—all of them normal or glycated (in diabetic case). The thickness was measured with a micrometer (accuracy about ±10  μm) as well as using the technique based on the delay of the re-reflected THz pulse. Using of the re-reflected THz pulse reduces the error in determining both the thickness and refraction to 1%. The accuracy of determining the thickness from the reflections is about 3  μm. As the pellets consist of biological crystals with an average size of about tens of micrometers, some surface roughness of the pellets remains. The blood plasma pellets were a little fragile; therefore, this method for measuring the thickness prevented possible damages.

The density of the pellets is calculated according to the following equation:

$$\rho_{o}=m\cdot {\left(\frac{\pi \cdot D^{2}}{4}\cdot d\right)}^{-1},$$ (1)

where m is the mass, D is the diameter, and d is the thickness of a pellet.

Nine pellets were made to study their optical characteristics using THz-TDS. At the first step, a pellet with a diameter of 13 mm was made, but it required a large amount of lyophilized plasma. In this regard, pellets with a diameter of 5 mm began to be produced. The measured thickness of pellets varied from 0.52 up to 1.81 mm and the calculated density varied from 1.000 up to $1.358\;{\mathrm{mm}}^{3}/\mathrm{mg}$. The pellets of samples from the control group of patients hereinafter “non-diabetic pellet” and the pellets of samples from the diabetic group of patients hereinafter “diabetic pellet.” Some macroscopic characteristics and calculated density of the pellets are summarized in Table 2.

**Table 2:** Macroscopic characteristics of blood plasma pellets and a moulding pressure.

Type of pellet	Diameter (mm)	Thickness (mm)	Weight (mg)	Density (${\mathrm{mm}}^{3}/\mathrm{mg}$)	Pressure (ton)
Diabetic	5	0.52	13.4	1.31	1.0
	5	0.76	15.8	1.06	0.5
	13	1.05	28.0	1.36	1.0
	5	1.12	25.9	1.17	0.5
	5	1.71	34.7	1.03	0.5
	5	1.81	37.0	1.00	0.5
Non-diabetic	5	0.85	17.5	1.05	0.5
	5	1.25	25.5	1.04	0.5
	5	1.79	35.1	1.00	0.5

**Fig. 1:**
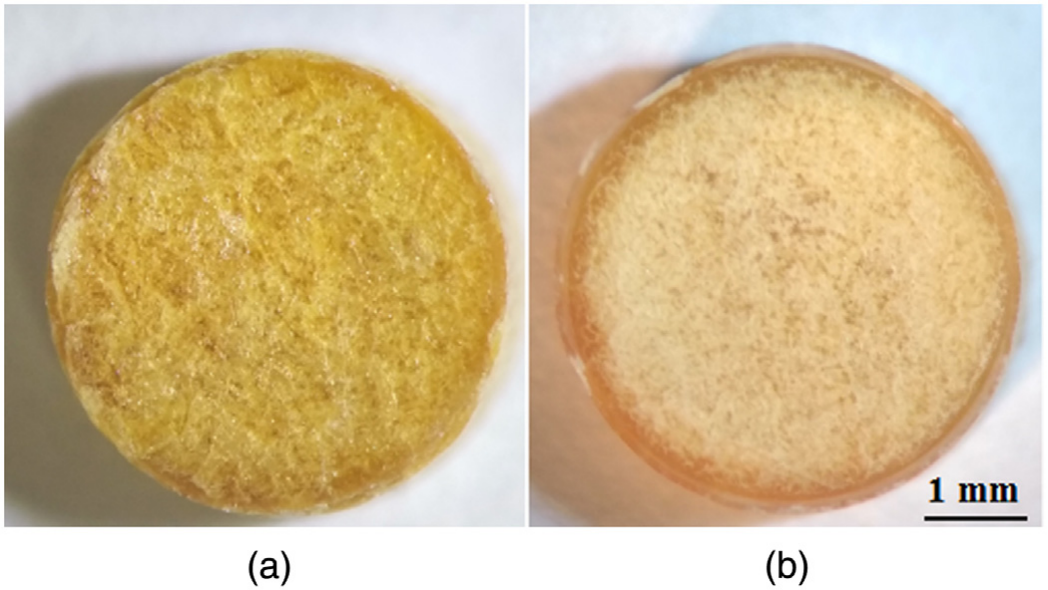
Photo of blood plasma (a) non-diabetic and (b) diabetic pellets.

The photos of pellets are presented in Fig. 1; the color of pellets is slightly different. Some physiological reasons may influence their color in visible electromagnetic range, for example, hemolysis (the release of blood cells content into plasma). Increased triglyceride concentration (lipids) may cause turbidity, which can be also visible.^30^

### 2.2 THz-TDS Experimental Setups

The samples were studied using THz-TDS in transmission mode, which was implemented on a custom-made spectrometer in St. Petersburg THz Laboratory, hereinafter “TDS-1,” and a custom-made spectrometer in Moscow Laboratory, hereinafter “TDS-2.”

TDS-1 spectrometer is equipped with a femtosecond Ti:sapphire laser, which was used to generate THz radiation and to detect it.^26^^,^^31^ The average wavelength was 800 nm, a pulse frequency of 80 MHz with an average power of 0.65 W, and an optical pulse length of 15 fs. The pump pulse passes through the delay line and excites an indium arsenide epilayer grown on a semi-insulator gallium arsenide wafer. For detecting the THz radiation, a 1-mm-thick (1 1 0) ZnTe crystal was used in the electro-optical sampling arrangement. The spectral range was 0.2 to 2.5 THz, which was mainly defined by the electro-optical sampling. At the optimal frequency of 1 THz, the signal-to-noise ratio (SNR) was $10^{4}$, which provided sufficiently reliable determination of optical characteristics of materials in this spectral range. The blood plasma pellets were placed on a holder moved by means of motorized positioners in the vertical and horizontal directions relative to the plane of incidence of the paraxial THz beam in the spectrometer. THz radiation was focused at nine different points on blood plasma pellets; the diameter of THz radiation spot was about 1.2 mm. To improve accuracy, the results were averaged.

TDS-2 spectrometer was equipped with a femtosecond Ti:sapphire laser with a central wavelength of 790 nm, a pulse frequency of 80 MHz, and turnable pulse duration 80 to 120 fs.^32^ The THz radiation was generated and detected using two photoconductive antennas, which were more efficient at lower frequencies. The frequency range of the spectrometer was 0.2 to 1.4 THz (SNR>10), at an optimal frequency of 1 THz, the SNR is $10^{3}$. The average power is 500 mW at the input of the THz emitter and 1  μW at the output. The THz signal was measured after transmission through blood plasma pellets. To improve the accuracy, the result was averaged over three independent experiments and for each type of measurement. The measurement of each pellet was performed in a special holder.

All measurements at TDS-1 and TDS-2 were made at room temperature (around 21°C±1°C).

### 2.3 Calculation the Absorption Coefficient and Refractive Index of Pellets

**Fig. 2:**
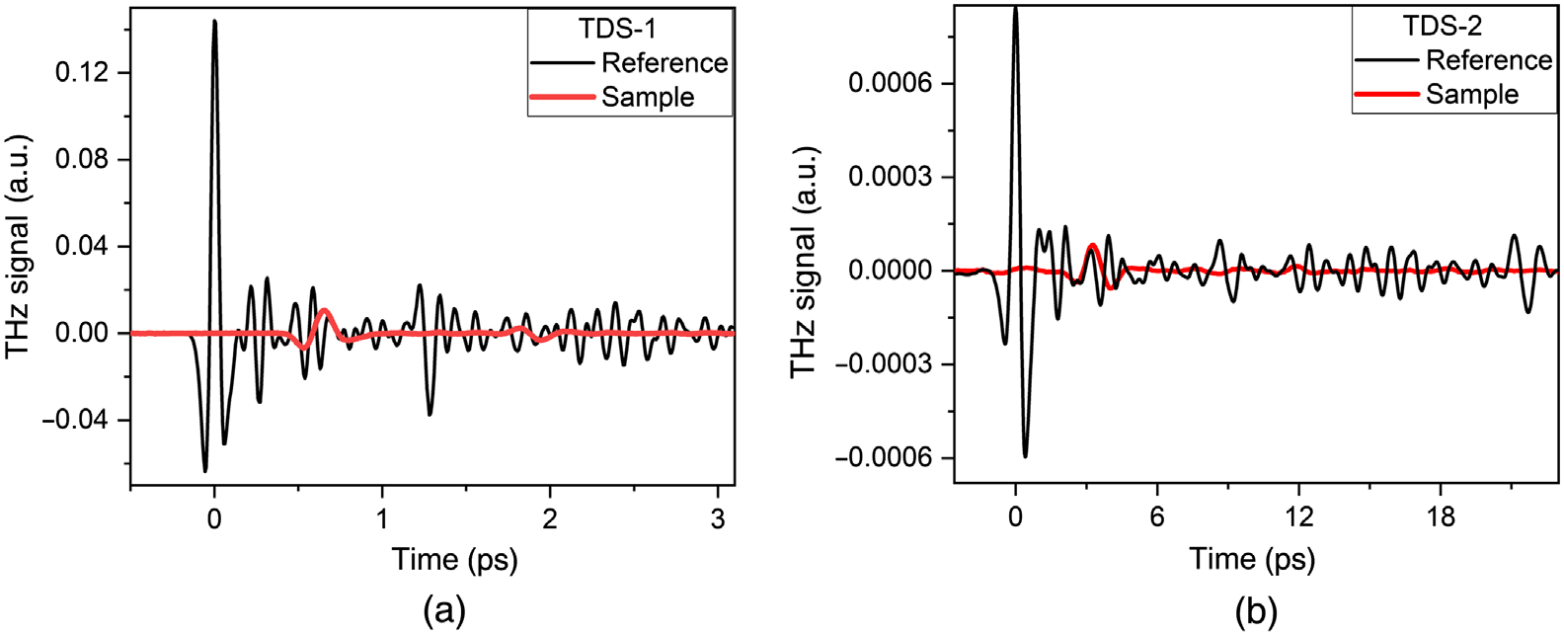
Typical waveforms of the reference signal and sample signal of two THz spectrometers: (a) TDS-1 and (b) TDS-2.

During the experiments using the TDS-1 and TDS-2 spectrometers, the wavefronts representing the dependence of the THz signal amplitude of the time delay between the pump pulse and the probe pulse were recorded. These oscillograms were obtained for THz pulses passing through the samples and through free space. Figure 2 shows an example of the THz pulses of each spectrometer transmitted through a blood plasma pellet and a reference signal.

To obtain the spectral components of the THz field, the fast Fourier transform (FFT) was used.^33^ Based on part of the FFT results (spectral amplitude and phase), the absorption coefficient and the refractive index were calculated.^34^

To calculate the optical properties of the samples, we used the frequency dependence of amplitude A(ω) and phase φ(ω). As a result, the following dependencies were calculated: $A_{\mathrm{ref}}(\omega )$, $\varphi_{\mathrm{ref}}(\omega )$, $A_{\mathrm{sam}}(\omega )$, and $\varphi_{\mathrm{sam}}(\omega )$,^35^ where ref is the reference and sam is the sample.

Since the phase spectral dependencies had different values at zero frequency, a correction was made by means of linear approximation and phase shift.

The calculations were based on the equations for electromagnetic radiation passing through the absorbing medium in the form of a pellet with thickness d.^34^ For the complex amplitudes of the THz field of the pulse passing through the sample and the free space, the following ratio can be obtained

$$\frac{E_{\mathrm{sam}}}{E_{\mathrm{ref}}}=\frac{4\cdot \overset{˜}{n}}{{\left(\overset{˜}{n}+1\right)}^{2}}\cdot \frac{1}{\left[1-\frac{{\left(\overset{˜}{n}-1\right)}^{2}}{{\left(\overset{˜}{n}+1\right)}^{2}}e^{i2\overset{˜}{k}d}\right]}\cdot e^{i\overset{˜}{k}d},$$ (2)

where $\overset{˜}{k}=\frac{\omega }{c}\overset{˜}{n}$ is the complex wave vector, ω=2πf is the angular frequency, $\overset{˜}{n}=n+i\varkappa$ is the complex refractive index, ϰ is the index of absorbance, and d is the sample thickness.

Since the time interval, in which only the first pulse is located, was chosen, and there are no THz pulses associated with the reflection of the THz wave from the sample surfaces, Eq. (2) can be reduced to

$$\frac{E_{\mathrm{sam}}}{E_{\mathrm{ref}}}=\frac{A_{\mathrm{sam}}}{A_{\mathrm{ref}}}\cdot e^{\left(\varphi_{\mathrm{sam}}-\varphi_{\mathrm{ref}}\right)}=\frac{4\cdot \overset{˜}{n}}{{\left(\overset{˜}{n}+1\right)}^{2}}\cdot e^{i\overset{˜}{k}d-\frac{\omega }{c}d},$$ (3)

where $A_{\mathrm{sam}}=|E_{\mathrm{sam}}|$, $A_{\mathrm{ref}}=|E_{\mathrm{ref}}|$.

Equation (3) indicates that there are no analytical expressions for n and ϰ from $\frac{A_{\mathrm{sam}}}{A_{\mathrm{ref}}}$ and $\varphi_{\mathrm{sam}}-\varphi_{\mathrm{ref}}$.

**Fig. 3:**
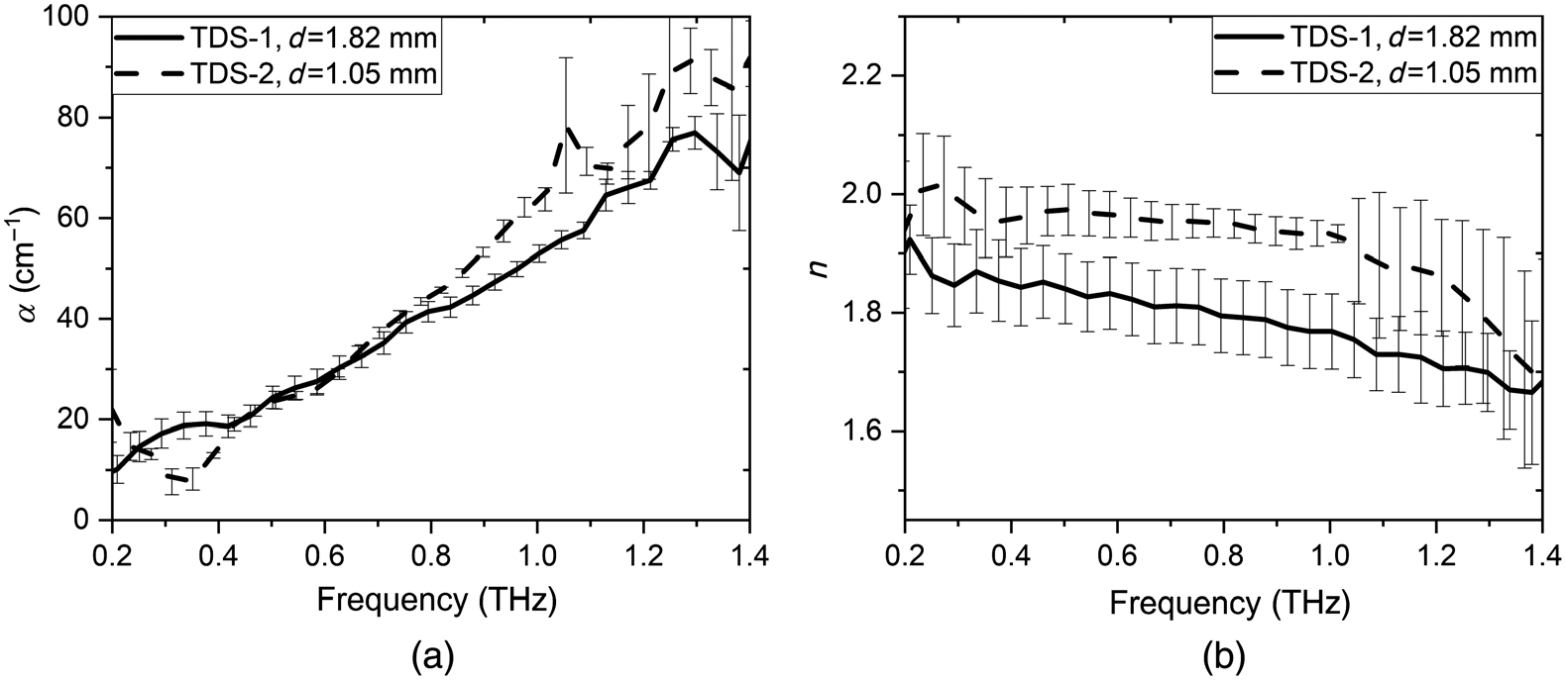
(a) Absorption coefficient and (b) refractive index of diabetic pellets with different thicknesses, measured on TDS-1 and TDS-2.

For the environments with absorption coefficient $\alpha <100\;{\mathrm{cm}}^{-1}$ [see Fig. 3(a)], the $\frac{\varkappa }{n}$ will be less than or of the order of 0.03 (f∼1  THz), which allows counting in the first approximation the value of the factor $\frac{4\cdot \overset{˜}{n}}{{\left(\overset{˜}{n}+1\right)}^{2}}$ equal to $\frac{4\cdot n}{{\left(n+1\right)}^{2}}$ in Eq. (3). Then for spectral amplitudes and phases, respectively, we obtain

$$\frac{A_{\mathrm{sam}}}{A_{\mathrm{ref}}}=\frac{4\cdot n}{{\left(n+1\right)}^{2}}\cdot e^{-\frac{\omega }{c}\varkappa d},$$ (4)

$$\varphi_{\mathrm{sam}}-\varphi_{\mathrm{ref}}=\frac{\omega }{c}nd-\frac{\omega }{c}d.$$ (5)

The refractive index is obtained from Eq. (5):

$$n=\frac{[\varphi_{\mathrm{sam}}-\varphi_{\mathrm{ref}}]\cdot 0.3}{360\cdot d\cdot f}+1.$$ (6)

If the refractive index is known, considering that $\frac{\omega }{c}\varkappa =\frac{\alpha }{2}$, it becomes possible to calculate the absorption coefficient of the sample from Eq. (4):

$$\alpha =\frac{2}{d}\;\ln[\frac{4\cdot n\cdot A_{\mathrm{ref}}}{{\left(n+1\right)}^{2}\cdot A_{\mathrm{sam}}}].$$ (7)

### 2.4 Data Acquisition and Processing Using PCA Analysis

The analysis of the absorption spectra of the pellets included a reduction of the dimension of the feature space using the principal component analysis (PCA)^36^^–^^38^ and predictive model construction using the support vector machine (SVM).^39^^–^^41^

The basic idea of PCA is to find the reduced number of new variables termed the principal components that are sufficient for the recovery of the initial variables, possibly with insignificant errors.^37^

The linear separability of non-diabetic and diabetic pellets was illustrated by an SVM with a linear kernel.^39^ The SVM constructs a hyperplane in feature space in order to maximize its distance from the class members. This method was originally developed to solve the problem of binary classification, but there are extensions to a multiclass case.

## 3 THz Optical Properties of Diabetic and Non-Diabetic Pellets

The averaged spectra of absorption coefficient and refractive index were obtained for diabetic pellets on two spectrometers TDS-1 and TDS-2. Figure 3 demonstrates that the absorption coefficient and refractive index of diabetic pellets with different thicknesses (1.05 and 1.81 mm) and weight (see Table 2), obtained on two spectrometers, are different.

Since the density of the pellets is also different, then it is necessary to exclude the effect of pressure during pellets pressing procedure and to normalize the THz optical properties (α and n) to the density of a pellet $\rho_{o}$:

$$\alpha_{\text{norm}}=\frac{\alpha }{\rho_{o}},$$ (8)

$$n_{\text{norm}}=\frac{\left(n-1\right)}{\rho_{o}}.$$ (9)

**Fig. 4:**
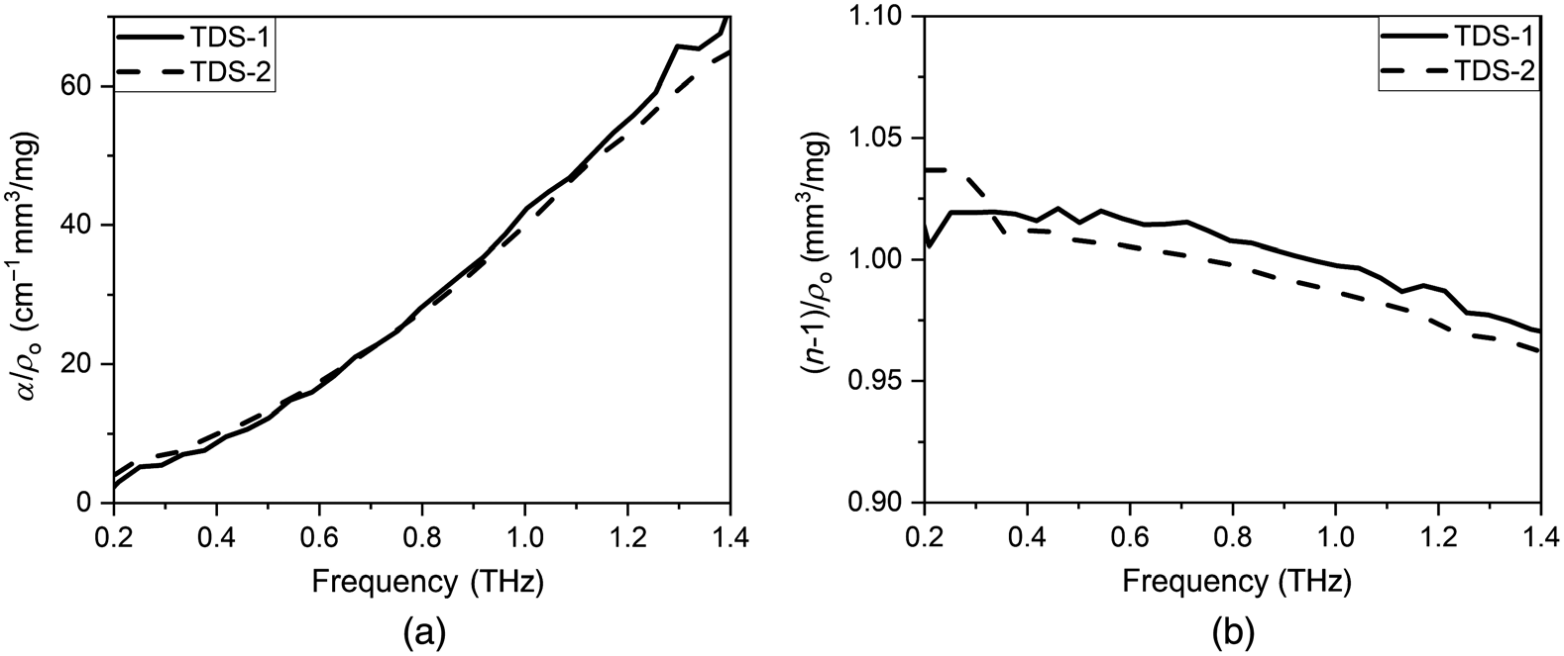
Density-normalized spectra of (a) absorption coefficient and (b) refractive index of diabetic blood plasma pellets with different thicknesses.

This assumption that α is proportional to $\rho_{o}$ is confirmed by the Bouguer–Lambert–Beer law and $\alpha /\rho_{o}$ has the meaning of molar extinction, i.e., the amount of absorption by one molecule. Thus $\alpha /\rho_{o}$ is the unique properties of the material in the THz frequency range. THz optical properties of diabetic and non-diabetic pellets measured on TDS-1 and TDS-2, obtained after their normalization, represented in Fig. 4. When normalized to the pellet density, the absorption coefficient and refractive index, obtained with each spectrometer, coincided on good accuracy.

**Fig. 5:**
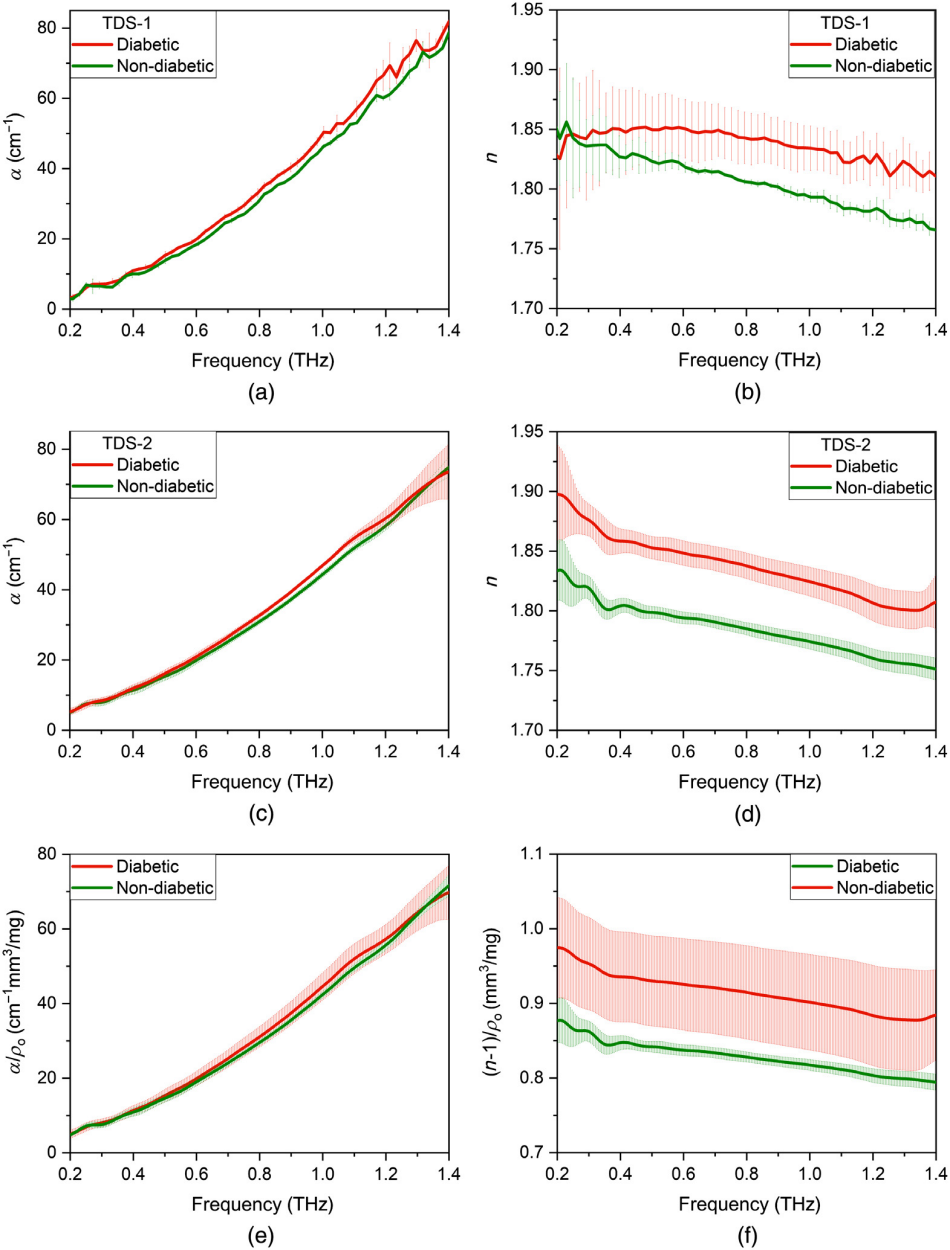
(a), (c) Absorption coefficient and (b), (d) refractive index of diabetic and non-diabetic pellets obtained on TDS-1 and TDS-2; averaged normalized (e) absorption coefficient and (f) refractive index of diabetic and non-diabetic pellets.

Figures 5(a)–5(d) present absorption and refractive index for diabetic and non-diabetic pellets obtained on TDS-1 and TDS-2 in the range of 0.2 to 1.4 THz. In the frequency range of 0.2 to 0.5 THz, the absorption coefficient of diabetic and non-diabetic pellets obtained on TDS-1 coincides with each other; the value amounts to 3.48 to $12.42\;{\mathrm{cm}}^{-1}$ [Fig. 5(a)]. In the frequency range of 0.5 to 1.4 THz, the absorption coefficient of diabetic pellets (13.9 to $82.0\;{\mathrm{cm}}^{-1}$) obtained on TDS-1 exceeds this indicator of the non-diabetic pellets (13.9 to $78.9\;{\mathrm{cm}}^{-1}$) on average by 0.38% to 15.37% [Fig. 5(b)]. At the same time, we see that the absorption coefficient obtained on TDS-1 almost corresponds to the absorption coefficient obtained on TDS-2 on the entire THz frequency range [Fig. 5(c)].

For both THz spectrometers, it corresponds that the refractive index of diabetic pellets exceeds this indicator of the non-diabetic pellets over the entire THz range [see Figs. 5(b) and 5(d)]. In the frequency range of 0.2 to 0.3 THz, the refractive index of non-diabetic pellets (1.842 to 1.856) exceeds this index of diabetic pellets (1.825 to 1.844), obtained on TDS-1 [Fig. 5(b)] on average by 0.65% to 0.93%. In the frequency range of 0.3 to 1.4 THz, the refractive index of diabetic pellets (1.810 to 1.851) exceeds this index of non-diabetic pellets (1.765 to 1.837), corresponding to TDS-1 [Fig. 5(b)] on average by 0.76% to 2.55%. For TDS-2, data correspond that the refractive index of diabetic pellets (1.800 to 1.897) exceeds this index of non-diabetic pellets (1.751 to 1.834) in the frequency range of 0.2 to 1.4 THz on average by 2.79% to 3.43% [Fig. 5(d)].

Subsequently, we averaged all data from two THz spectrometers and normalized each sample to its density. Figures 5(e) and 5(f) presents the averaged density-normalized absorption coefficient and refractive index of diabetic and non-diabetic pellets obtained on TDS-1 and TDS-2. Over the entire THz frequency range, the normalized refractive index of diabetic pellets exceeds this index of non-diabetic pellets on average by 9% to 12% [Fig. 5(f)]. It is clearly seen in Fig. 5(e) that the normalized absorption coefficients of diabetic and non-diabetic pellets coincide with each other over the entire THz frequency range.

The experimentally observed effect corresponds to the trend described by the second group of researchers of liquid sugars (see Sec. 1). They supposed that the absorption intensities increase in both aqueous glucose solutions and aqueous fructose solutions with the concentrations of sugars increasing.^19^^,^^23^^,^^24^ As a result, we can analyze the graphs (Fig. 5) on the base of the biochemical parameters of liquid blood plasma obtained in this work (see Table 1). Table 1 shows that the concentration of glucose, triglycerides, and glycated hemoglobin in the samples of a patient with diabetes increases 1.5, 2.0, and 2.3 times, respectively. Therefore, the differences in the indicated biochemical parameters of plasma samples can be observed by their THz properties. It was discovered that the curve shape of the diabetic and non-diabetic pellets coincides with the curve of lyophilized albumin pellets described in Ref. 42. This effect can be explained by the fact that after plasma lyophilization, most of its composition consists of various proteins, where 55% to 65% is albumin. The normalized optical properties have no pronounced spectral features which can be explained by the absence of spectral absorption lines of amorphous glucose (lyophilized glucose) in the THz spectra.^27^

PCA was performed on the experimentally obtained TDS-1 and TDS-2 absorption spectra of the blood plasma non-diabetic and diabetic pellets using SVM. In total, 25 absorption spectra obtained on TDS-1 and 30 absorption spectra obtained on TDS-2 were taken for analysis in the spectral range 0.2 to 1.4 THz. Absorption spectra are the ratio between the amplitude of the sample $A_{\mathrm{sam}}$ and the amplitude of the reference signal $A_{\mathrm{ref}}$. PCA for TDS-2 data revealed that the first two principal components (PC1 and PC2) contain most of the explained variance (almost 90%), i.e., PC1 (65.8%) and PC2 (24%) respectively. The loadings matrix analysis shows that the most informative feature for PC1 is 0.25 THz, and 0.3 THz for the PC2. The first two principal components for TDS-1 data, i.e., PC1 and PC2 contain most of the variance (>94%) with a distribution of 85.70% and 6.27%, respectively.

**Fig. 6:**
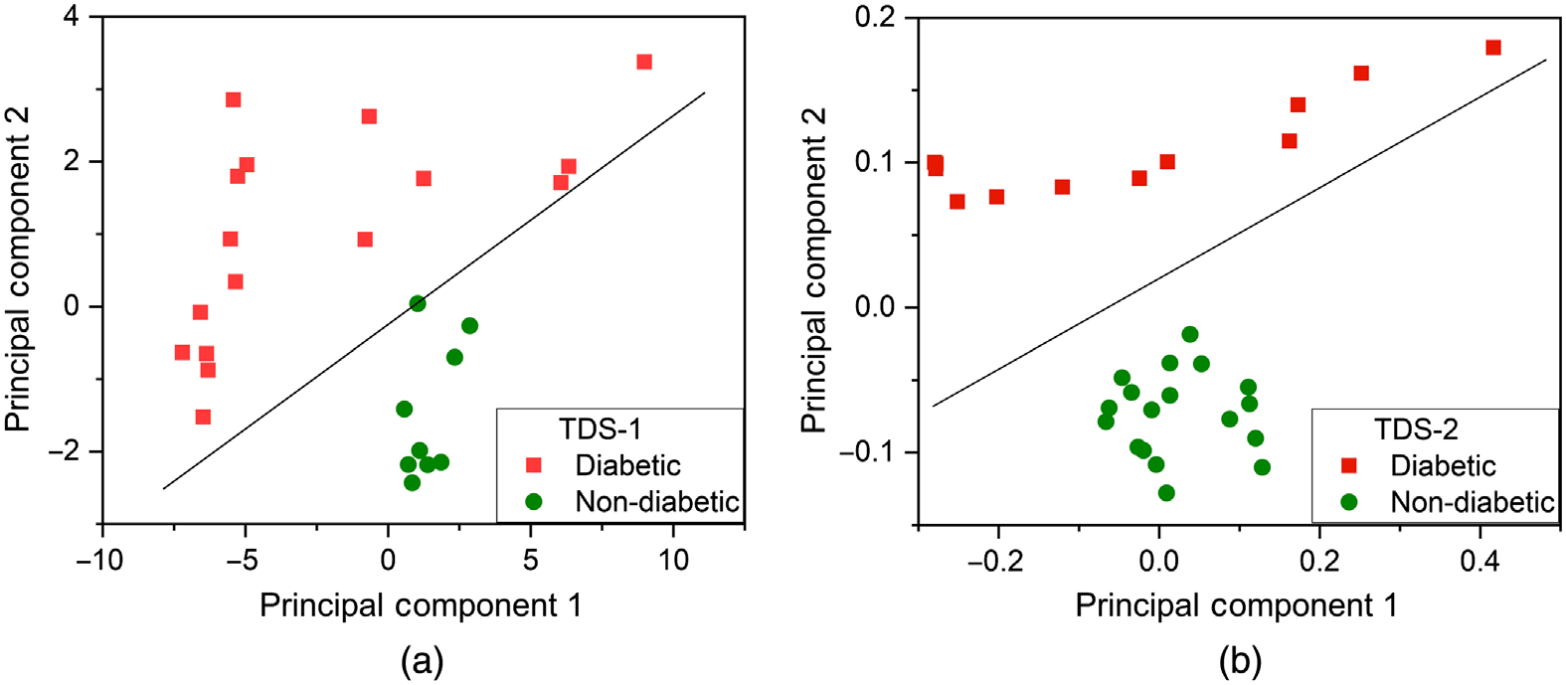
Projection of absorption spectra on the subspace of the first and second principal components obtained on (a) TDS-1 and (b) TDS-2.

The linear separability of non-diabetic and diabetic groups of samples was illustrated by SVM with a linear kernel (C=1.0, decision function is one-versus-rest). The results are presented in Fig. 6.

As it can be seen from Fig. 6, the non-diabetic and diabetic groups of the THz spectra for both THz spectrometers have a rather compact distribution in the principal component space that confirms a small intergroup variation of raw spectral data. Both models for TDS-1 and TDS-2, absorption spectra have good separability, which indicates a possibility to construct predictive models if the number of the analyzed samples will be increased. This illustrates the potential ability in clinical medicine to construct a predictive rule by supervised learning algorithms after collecting enough experimental data.

## 4 Conclusion

Tracking the dynamic changes in the level of glucose, lipids, biomarkers, and various hormones in different chronical diseases requires storing the biomaterial in specially designed storage facilities. These days, long-term storage (for 2 to 5 years), in particular, for plasma samples, requires placing several 1.5 ml Eppendorfs in freezers maintaining a temperature below −80°C. When evaluating each laboratory parameter, from 15 to 100  μl of plasma are consumed and lost irreversibly. As a result, the stored volumes are often insufficient to estimate some newly identified biomarkers several years after the storing, and valuable scientific information becomes unavailable. The creation of fundamentally new approaches to storing biomaterial and estimation their various parameters, without irreversible loss of biomaterial, is a pressing challenge in clinical medicine.

In this work, a technology for studying and storing blood plasma using the lyophilization of blood plasma has been presented. The lyophilization method allows getting dry tissue without losing their structural integrity and biological activity. Dried blood plasma is a sponge consisting of biological crystals. The dry mixture of blood plasma crystals has been pressed into a flat pellet in a steel press mold with a diameter of 5 mm. The pellets are the tablets pressed from small-fractional protein crystals. Each crystal of pellets contains a certain percentage of lipids (triglycerides), proteins (albumin), and fibrinogen—all of them are normal or glycated (in diabetic case).

A review of the literature has shown that, over the past five years, THz spectroscopy has been actively studied for the analysis of model sugar solutions. It can be used for dynamic control of glycation processes; it is sensitive to the type of sugar involved in glycation and is sensitive to the pH value during glycation. This study was performed with blood plasma of conditionally healthy participants and patients with type 2 diabetes mellitus. The analysis of diabetic and non-diabetic blood plasma has been implemented using traditional biochemical methods used in a medical center (in liquid form) and using THz-TDS (in tablet form) in the range of 0.2 up to 1.4 THz.

Since the density of the pellets has been different, it becomes necessary to exclude the effect of pressure during pellets pressing and to normalize the spectra to the pellet density. The assumption that absorption coefficient is proportional to the pellet density is confirmed by the Bouguer–Lambert–Beer law, and the ratio of these parameters has the meaning of molar extinction. When normalized to the pellet density, the absorption coefficient and refractive index obtained with two different THz spectrometers coincides with good accuracy. Over the entire THz frequency range, the normalized refractive index of diabetes pellets exceeds this indicator of non-diabetic pellet on average by 9% to 12%.

Analysis of the THz absorption spectra of pellets includes a reduction of the dimension of the feature space using the PCA. The groups of the THz spectra have a rather compact distribution in the principal component space. The satisfactory linear separability of the non-diabetic and diabetes groups is illustrated by the SVM with a linear kernel. The next step in multivariate data analysis should be aimed at supervised learning. It allows assessing patient’s condition. The corresponding efforts will be associated with a collection of sufficient data from patients with known conditions.
